# Bibliometric Analysis of Sustainable Practices in Hotels and Homestays: Trends, Themes, and Future Research Directions

**DOI:** 10.12688/f1000research.184973.1

**Published:** 2026-07-06

**Authors:** Biswajit Ghose, Pankaj Kumar Saha, Pankaj Kumar Tyagi, Premendra Kumar Singh, Asokan Vasudevan, Renee Shiun Yee Chew, Shamsiah Banu Mohamad Hanefar

**Affiliations:** 1 Department of Commerce, Tezpur University, Napaam, Assam, India; 2 University Institute of Tourism and Hospitality Management, Chandigarh University, Sahibzada Ajit Singh Nagar, Punjab, India; 3 Centre for Distance & Online Education, Sharda University, Greater Noida, Uttar Pradesh, India; 4 Faculty of Business and Communications, INTI International University, Nilai, Negeri Sembilan, Malaysia; 5 Faculty of Education and Liberal Arts, INTI International University, Nilai, Negeri Sembilan, Malaysia

**Keywords:** Sustainability, Homestays, Hotels, Bibliometric Analysis, Thematic Evolution, Energy Efficiency, Nature Inclusive

## Abstract

**Background:**

Sustainability has emerged as a critical concern in the hospitality sector, however there is a lack of a systematic review of the academic literature on sustainability in hotels and homestays. The rise of environmental concerns, responsible tourist practices, and the growing importance of sustainable hospitality have significantly expanded the academic interest in this area of study. This study provides a bibliometric review of the literature on sustainability in hotels and homestays to identify patterns of growth, dominant themes, and research gaps to guide future research and policy.

**Methods:**

We retrieved 1,932 articles published between 1998 and 2025 from the Web of Science Core Collection. Bibliometric analyses were conducted using Bibliometrix/Biblioshiny and VOSviewer to examine yearly publication trends, prolific journals and authors, collaboration networks, keyword co-occurrence, and thematic evolution through strategic thematic mapping.

**Results:**

Publication volume rose markedly after 2016, reflecting the fast growth of academic attention. Core topics clustered around impact, satisfaction, tourism, management, and performance. Thematic mapping classified clusters as Basic Themes (impact–satisfaction–management), Motor Themes (attitudes–knowledge), Niche Themes (business models–consumer acceptance), and Emerging/Declining Themes (energy consumption). Analysis also revealed growing emphasis on heritage, innovation, and technology-enabled sustainability. Notable gaps persist i.e. rural homestays remain underexplored, corporate social responsibility (CSR) integration is limited, and stakeholder collaboration is insufficiently addressed. While collaboration networks reveal clusters of authors, there is scope for greater interdisciplinarity and cross-regional relationships.

**Conclusion:**

This bibliometric review synthesises the intellectual structure of sustainability research in hotels and homestays, highlighting established and emergent topics while pinpointing substantive knowledge gaps. The findings offer a roadmap for researchers to diversify contexts (especially rural homestays), integrate CSR and stakeholder perspectives, and pursue interdisciplinary methods. For policymakers and practitioners, the study recommends developing inclusive, technology-enabled sustainability frameworks that better connect heritage conservation, innovation, and community engagement.

## Introduction

Sustainability has become an issue and a key goal in the hospitality industry as environmental, economic, and socio-cultural challenges of global development and consumption have increased ( Buffa et al., 2018; Gomezelj, 2016). As an important element in the accommodation industry, hotels and homestays play a critical role in developing and exercising sustainable practices at the operational, business, and community interface ( Raub et al., 2021). In recent 20 years, sustainability has progressed beyond a speciality issue to achieve the status of a popular research topic, shaping academic studies in such domains as energy efficiency, resource conservation, customer satisfaction, employee engagement, and corporate social responsibility ( Yang et al., 2023). Among these accommodation types, homestay businesses have become more prevalent because the kind of accommodation has a high connection with the local culture, rural development and authentic experiences of the visitors. It is a part of the whole immersion of the country, which the guests are integrated into, as part of the local customs and traditions in homestays ( Basak et al., 2021). Moreover, they are the means of community empowerment/grassroots economic development, particularly in rural or marginal areas ( Kontogeorgopoulos et al., 2015). Although researchers have been showing an interest in homestays, the academic literature on them is still barely developed in comparison to the research in the field of general hospitality, particularly involving sustainability and a long-term effect ( Janjua et al., 2023).

Moreover, the sustainability topic has been well recognised by the hospitality industry, but the rate of adoption and diffusion of sustainable measures has been uneven, and the systematic review of the research on the topic is rather scarce ( Acampora et al., 2022). The recent literature has insisted on the necessity to chart the progress of sustainability in the hospitality sector, on which themes have been studied most, which researchers or nations have been most prolific, and to what emerging themes they should look moving forward ( Pasanchay & Schott, 2021). The bibliometric analysis would provide a sound methodological framework to address these questions by quantifying a systematic way of inquiries about the intellectual, conceptual, and even the social framework of a knowledge domain in a rigorous way ( Donthu et al., 2021). Whereas previous literature has used bibliometric or systematic reviews of literature in other wider fields of sustainable tourism and hospitality, there has been few literature that uses bibliometric or systematic literature analysis to study the interrelation of sustainability and accommodation, particularly in the comparison between hotels and homestays ( De Bruyn et al., 2023). In addition, the paradigms that interpret the nature, development, and thematic progression of sustainability research in the hospitality sector are often fragmented and disjointed ( Acampora et al., 2022). While prior studies have explored sustainability within tourism and hospitality through bibliometric and systematic reviews, these studies typically emphasise broader domains and overlook detailed comparisons between accommodation types like hotels and homestays ( Yang et al., 2023). There is, therefore, a necessity for a qualitative bibliometric study that brings conceptual clarity to the development of sustainability as an academic interest, specifically in the context of hotel and homestay accommodations ( Donthu et al., 2021).

This paper aims to address this gap by performing a bibliometric analysis of 1,932 peer-reviewed articles within the Web of Science Core Collection, which spans the years 1998 to 2025. Using some bibliometric tools like Bibliometrix, Biblioshiny, and VOSviewer, the article traces the structure, changes, and evolution of the research on sustainability in this field. Considerations taken are conceptual (keywords, themes, research clusters) and intellectual (authors, journals, institutions), as well as thematic change over time. ( Aria & Cuccurullo, 2017).

To direct the research, the following research questions are answered:

**RQ1:** What is the current state and temporal evolution of research on sustainability in hotels and homestays?

**RQ2:** Who are the most influential authors, institutions, countries, and journals contributing to sustainability research in hotels and homestays?

**RQ3:** What are the major thematic clusters and conceptual structures in the literature on sustainability practices in hotels and homestays?

## 3. Methodology

### 3.1. Search strategy

To explore how sustainability is being studied in hotels and homestays, we searched for research papers in the Web of Science Core Collection, one of the most trusted academic databases ( Mongeon & Paul-Hus, 2016). Based on earlier research guidelines ( Marzi et al., 2025), we created a search using important terms such as “sustainability,” “sustainable practices,” “green practices,” “eco-friendly practices,” and “environmentally friendly practices”, combined with the words “hotel” and “homestay” ( Donthu et al., 2021). We searched for these terms in the titles, abstracts, and keywords of the papers ( Zupic & Čater, 2015). The initial search gave us 2,796 results. Then, we filtered the results to include only papers written in English and limited them to research articles, review papers, and early access articles ( Gusenbauer & Haddaway, 2020). We also focused on papers published in top journals like *Sustainability*, *Journal of Sustainable Tourism*, *International Journal of Hospitality Management*, and *International Journal of Contemporary Hospitality Management.* After applying these filters, we ended up with 1,932 relevant papers, which we used for our analysis. This process enabled us to collect high-quality and meaningful research on sustainability in hotels and homestays.

### 3.2. Data source

The bibliometric dataset used in this study includes a total of 1,932 publications retrieved from the *Web of Science Core Collection*, covering the period from 1998 to 2025. These documents focus on sustainability-related topics within the context of hotels and homestays. The current set has a total of 48,279 citations with an average of 24.81, and thus it proves a significant interest on the scholarly level in the given area ( Donthu et al., 2021). Moreover, the dataset shows a strong academic impact with an h-index of 100, which means that there are at least 100 articles published in it that have been cited at least 100 times ( Hirsch, 2005). This strong and substantial literature leads to a good basis of bibliometric analysis and enables the assessment of the main research directions, key contributors, and major thematic areas within the sustainable practices subject in hotels and homestays ( Aria & Cuccurullo, 2017; Zupic & Čater, 2015).

### 3.3. Data cleaning and inclusion criteria

A systematic filter procedure was used based on the first saved search to question the quality of the dataset and its relevance ( Donthu et al., 2021; Zupic & Čater, 2015). Publications in languages other than English were excluded to provide linguistic homogeneity in the analysis and interpretation of data ( Mongeon & Paul-Hus, 2016). The type of documents was limited to peer-reviewed articles, review papers, and early access publications, which guaranteed the inclusion of scholarly work and academic rigour ( Gusenbauer & Haddaway, 2020). Moreover, to concentrate the research work on high-quality and relevant sources, the sample was narrowed down to the publications by the most influential journals, i.e., *Sustainability*, *Journal of Sustainable Tourism*, *International Journal of Hospitality Management*, and *International Journal of Contemporary Hospitality Management.* These filters ended up with a final set of relevant publications numbering 1,932.

### 3.4. Tools used

To perform the analysis of the dataset, we applied the *Bibliometrix* R package, a wide-ranging, free, and open-source tool, which is developed specifically to work with bibliometric studies ( Aria & Cuccurullo, 2017). This set made it possible to assess the literature quantitatively, as well as conceptually map it. The *Bibliometrix* interface was deployed using the *Biblioshiny* interface since it is a convenient and accessible web application that improves usability. It enables non-programmer researchers to run large-scale bibliometric studies and easily produce meaningful graphic representations ( Aria & Cuccurullo, 2017; Donthu et al., 2021). Besides *Bibliometrix*, we also integrated *VOSviewer*, a software package for building and visualising bibliometric networks (co-authorship, keyword co-occurrence, and citation networks) ( Van Eck & Waltman, 2010). The interaction of *Bibliometrix* and *VOSviewer* is sufficiently common in bibliometric literature as one of the robust and compatible integrations ( Marzi et al., 2025). All of these tools built the analytical core of the research and allowed exploring the publication patterns, intellectual networks, thematic development, and the collaboration patterns of the authors in depth. In addition, to ascertain transparency and rigour during the data selection procedure, we developed a *PRISMA 2020* flowchart ( Fig. 1) detailing the inclusion criteria and exclusion criteria carried out during the procedure of refining datasets ( Page et al., 2021). This is a combination of analysis tools and forms a comprehensive, very reproducible, and methodologically strong bibliometric analysis.

**Figure 1.**
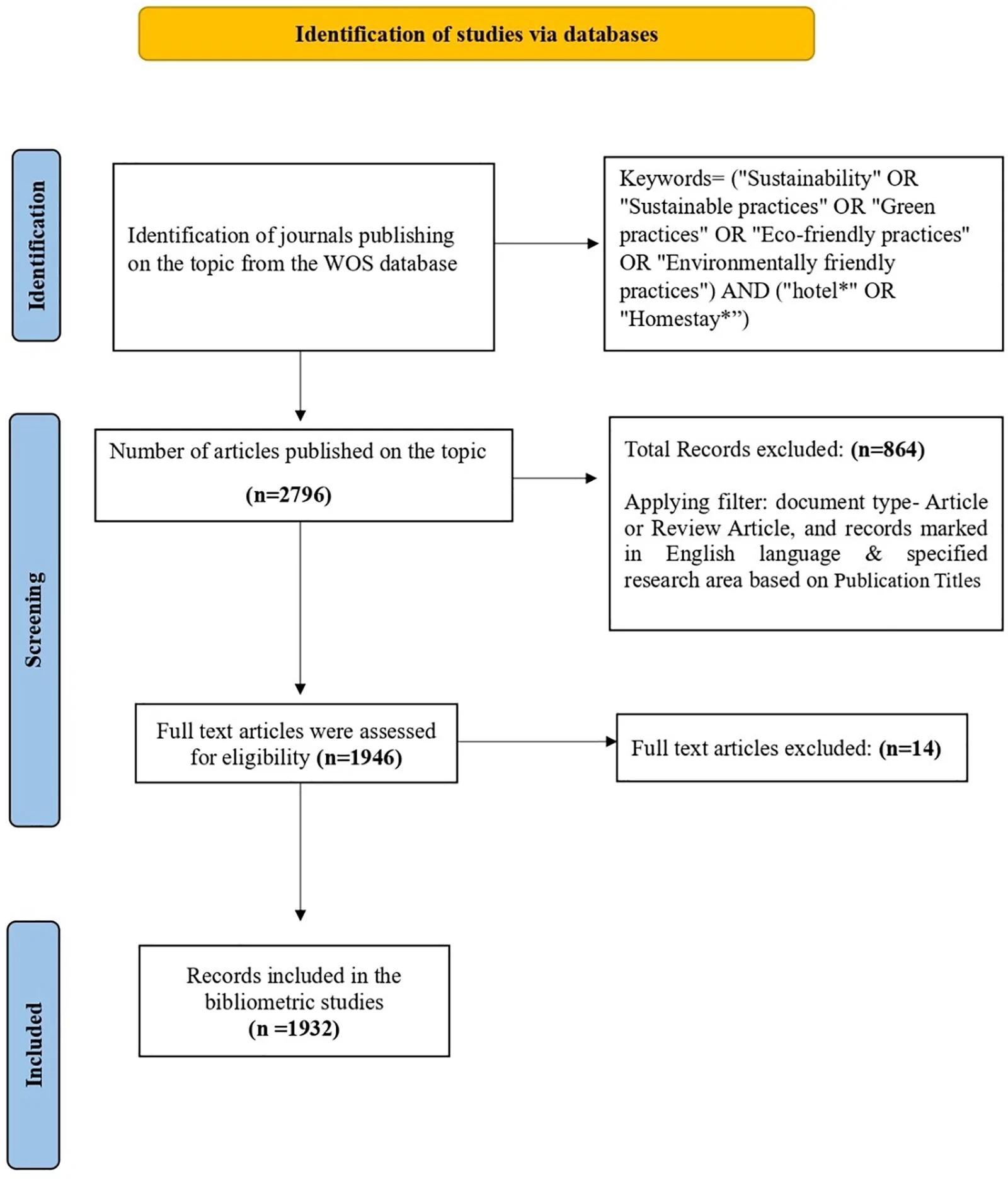
A PRISMA flowchart for the literature screening process.

### 3.5. Bibliometric approach

The present study relies on the bibliometric approach, which implements quantitative techniques to work with bibliographic and citation data. Bibliometric reviews examine large quantities of published data, unlike the traditional systematic literature reviews, which mostly emphasise qualitative discussion. This technique assists in determining the research trends, patterns, and relationships in a general area of investigation. The study uses a quantitative bibliometric approach to map the intellectual and conceptual framework of sustainability studies of hotels and homestays. Bibliometric analysis is commonly accepted as a supplementary method of systematic literature reviews and meta-analysis, as it provides strong evaluation using citation-based metrics, authorship, institutional impact, and geographical patterns ( Donthu et al., 2021; Zupic & Čater, 2015). Co-citation analysis is a bibliometric method that is frequently used in research ( Singh & Chaurasia, 2022). It also looks at the dependence of documents by looking at the frequency of co-occurrence of two articles in subsequent research ( Shiau et al., 2023). Two publications being mentioned in the same paper are said to be co-cited, which points to a close intellectual relationship between them ( Aria & Cuccurullo, 2017). This is a technique to determine key research trends and the general knowledge organisation of a discipline. Hence, in this study, co-citation analysis was used to investigate the emergence of sustainability research in the hotels and homestay research ( Arici et al., 2022).

The data set is composed of journal articles, books, book chapters, review papers, proceedings, and editors. Three of them were used: citation analysis, which identifies the influence of works that shape the field ( Gundolf & Filser, 2013; Hota et al., 2020; Zupic & Čater, 2015); co-citation analysis, which reveals the foundational knowledge by comparing documents cited concurrently ( Donthu et al., 2021; Sarin et al., 2020; Small, 1973); and co-word analysis, which explores relationships among keywords to reveal thematic structures and emerging trends ( Callon et al., 1983; Zupic & Čater, 2015).

### 3.6. Timeframe and dataset summary

The final list offered 1,932 articles published yearly between 1998 and 2025 that go beyond three decades of academic work in the sustainability area of hotels and homestays. The publishing materials were acquired through the Web of Science Core Collection, which is a credible academic publication that uses comprehensive indexing. A total of 48,279 citations has been obtained in the dataset, with an average of 24.81 per document. The computed value of the h-index of the data counts was 100 this implies it has had a huge influence and that there has always been a topicality with academic concern in the field of study. The data refers not only to the amount of scholarly production but also to the variety of research topics and authors, which creates a strong background for bibliometric analysis.

Table 1 depicts general information about the dataset. The sources published the documents between 1998 and 2025 in a range of 18 sources. The modal age of published documents is 4 years, and an average of 24.67 references is directed to each published document. This data contains 96,650 references and 9,152 unique keywords, 2,957 of which were Keywords Plus and 6,195 were author-supplied. These documents are published by 5,062 authors; 119 works are single-authored. It can be estimated that about 42.6 percent of the works are the products of international cooperation, which demonstrates high worldwide scholarly interest in the sustainability of hotels and homestays.

**Table 1.** Main data information.

Description	Results
Timespan	1998:2025
Sources (Journals, Books, etc)	18
Documents	1932
Annual Growth Rate %	20.02
Document Average Age	4
Average citations per doc	24.67
References	96650
DOCUMENT CONTENTS	
Keywords Plus (ID)	2957
Author’s Keywords (DE)	6195
AUTHORS	
Authors	5062
Authors of single-authored docs	107
AUTHORS COLLABORATION	
Single-authored docs	119
Co-Authors per Doc	3.78
International co-authorships %	42.6

## 4. Results and discussion

### 4.1. Descriptive inferences

***4.1.1 Annual publication trend***

Figure 2 shows the chronological path of academic publications with regard to sustainability in hotels and homestays. Its first contribution came in 1998, but long-term academic interest did not arise until 2005. Publications gradually gained an upward slope over the next ten years, and then a significant growth began in 2016. There was a significant increase between 2018 and 2021, and 298 articles were published in 2021, a record. This growth persisted up to 2022, 2023, and 2024, with the number of publications being 285, 272, and 255, respectively, signifying the sustainable scholarly interest.

**Figure 2.**
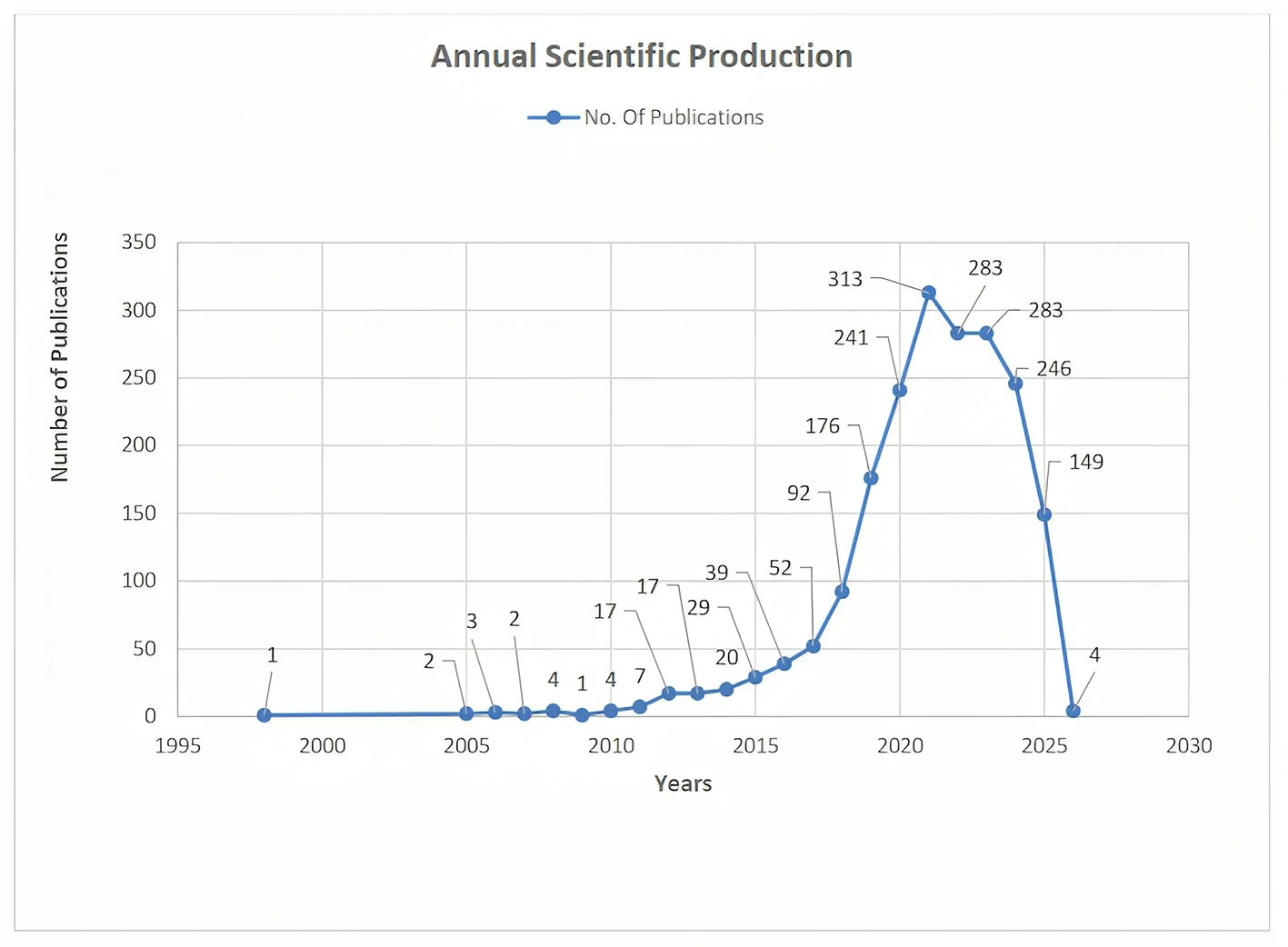
Scientific growth in sustainability research in homestays and hotels.

***4.1.2 Citation trend***

Citation measures further explain the impacts of such research. The earlier articles (2005 and 2007) recorded very high average citations per new publication (169.5 and 224.5, respectively), which shows their continuous impact. Similarly, 2012, 151.06 citations/article and a 10.79 citation rate also shaped up as a very fruitful and influential year. Because of their shorter time to disseminate, the average citations per publication of more recent articles also decrease, but with their presented constant activity since 2020, as shown in Figure 3, indicates that sustainability as a research topic in hotels and homestays will be relevant and generate new studies.

**Figure 3.**
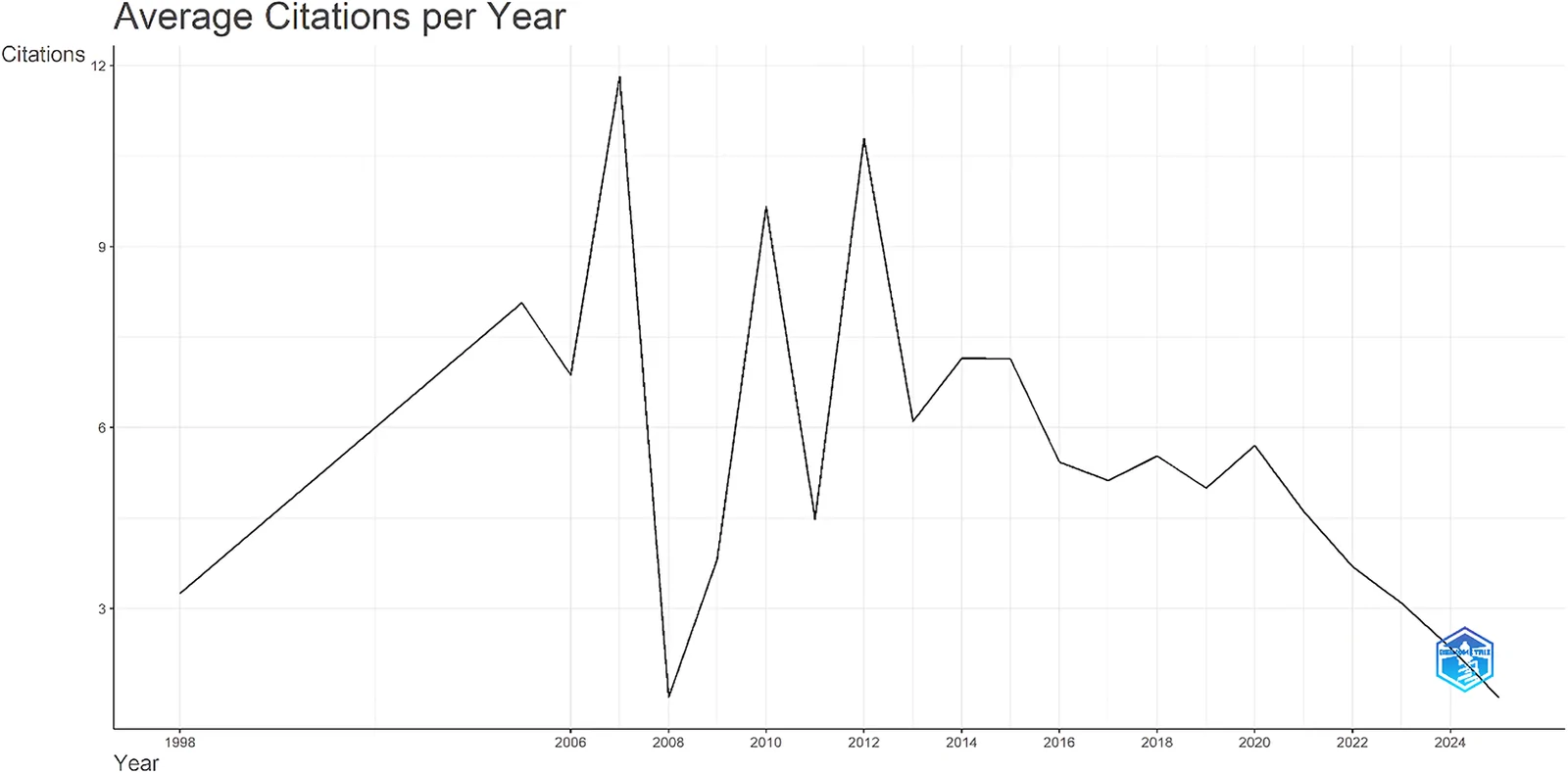
Annual average number of citations in sustainability research in homestays and hotels.

### 4.2. Influential sources and contributors

***4.2.1 Top journals***

Table 2 brings the ten most cited journals on sustainability in hotels and homestays that give an idea of the ten most active and influential publication sources in the area of sustainability in the hotel and homestay sector. (1) When it comes to sustainability, directly linked journals are connected with tourism. Sustainability leads with 1,227 works, and it makes up 63.07 percent of the total publications. Journal of Sustainable Tourism comes next with 130 (6.68 percent) articles. Although Sustainability is a universally scoped, multidisciplinary, open-access journal, the Journal of Sustainable Tourism is subscription-based and is considered a highly recognised publication. They dominate because they are in line with the major themes of the study. (2) The journals have a huge difference in the volume of publication. The two most popular journals alone add up to almost 70 per cent of the cumulative literature, and the other eight journals make up the other 30 per cent. Minor but important roles are played by journals such as International Journal of Hospitality Management (5.65%), International Journal of Contemporary Hospitality Management (4.47%), and Journal of Cleaner Production (3.03%). (3) Most of the mentioned journals have high impact factors, which means that they are academically influential. In particular, the impact factor of seven of these journals is higher than 5, and four of them are more than 10. Among the most prominent ones are Journal of Hospitality Marketing Management (20.9), Tourism Management (12.4), Journal of Cleaner Production (10.7). These scores not only indicate the effectiveness and topicality of the studies published in these sources but also provide a way for effective publication for the following researchers.

**Table 2.** Top journals in the context of sustainability research in homestays and hotels.

Ranking	Journal	5-Year IF	Count	Percentage
1	Sustainability	3.6	1,227	63.05%
2	Journal of Sustainable Tourism	9.9	130	6.68%
3	International Journal of Hospitality Management	10.3	110	5.65%
4	International Journal of Contemporary Hospitality Management	9.2	87	4.47%
5	Journal of Cleaner Production	10.7	59	3.03%
6	Environment Development and Sustainability	4.5	48	2.47%
7	Tourism Management	12.4	46	2.36%
8	Current Issues in Tourism	6.7	45	2.31%
9	Journal of Hospitality and Tourism Management	7.8	31	1.59%
10	Journal of Hospitality Marketing Management	20.9	24	1.23%

***4.2.2 Top categories***

Table 3 represents the leading subject areas in the study of sustainability in hotels and homestays. All in all, the evidence refers to a highly developed interdisciplinary background that is interested in, e.g., environmental science, tourism, management, business, and social sciences. In particular, the dominance is attributed to Green Sustainable Science and Technology, Environmental Sciences and Environmental Studies with 75.23, 68.55 and 65.42 per cent of publications, respectively. These findings indicate that the sustainability research in this area is predominantly environmental research. Significance of Hospitality, Leisure, Sport, and Tourism is also important (30.78%), which implies direct participation of the hospitality industry in the challenges of sustainability. Other contributions are broken down into the following categories: Management (10.64%), Environmental Engineering (3.03%), Business (1.23%), Sociology (0.77), and Multidisciplinary Sciences (0.67). Such diversity of categories justifies the interdisciplinary nature of the sustainability research within the framework of hospitality and tourism, where the views of science, business, and social sciences are offered to answer extremely multifaceted issues of sustainability ( Niñerola et al., 2019).

**Table 3.** Publication category statistics.

Ranking	Category	Count	Percentage
1	Green Sustainable Science Technology	1,464	75.23%
2	Environmental Sciences	1,334	68.55%
3	Environmental Studies	1,273	65.42%
4	Hospitality Leisure Sport Tourism	599	30.78%
5	Management	207	10.64%
6	Engineering Environmental	59	3.03%
7	Business	24	1.23%
8	Sociology	15	0.77%
9	Multidisciplinary Sciences	13	0.67%

***4.2.3 Country collaboration***

Figure 4 provides the map of the country collaboration, which introduces the correlations between the international research on collaboration within the domain of sustainability in hotels and homestays ( Aria & Cuccurullo, 2017). The darker colours are a scale of more publications, and the light colours are a scale of fewer publications. It is worth mentioning that the United States, China, the United Kingdom, Australia, and India have a high degree of interconnection, and this demonstrates high cooperation between the regions. Such a high interest in India in these international publications, as well as its increasing interest in the sustainability of hospitality research, confirms that sustainability in hospitality is a global challenge and a modern, developing field in the Indian context ( Blanco-Moreno et al., 2025).

**Figure 4.**
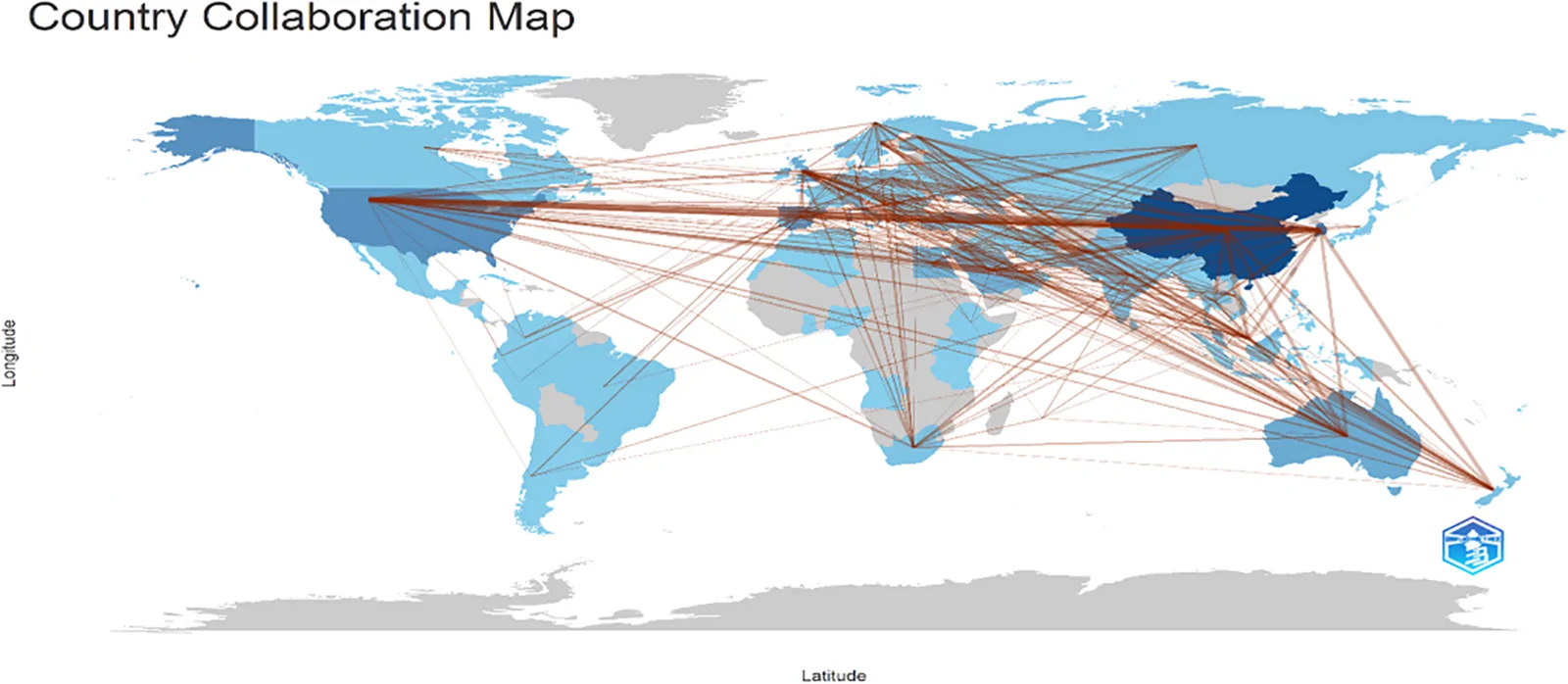
Country collaboration map in sustainability research in homestays and hotels.

***4.2.4 Most cited articles***

Table 4 gives the list of most frequently cited articles that have contributed greatly to the discussion of sustainability in hotels and other tourism settings. The listed studies are regarded as significant work because of the high number of citations. Jiang & Wen, (2020) received 495 citations on the effect of COVID-19 on hotel marketing and management, where crisis situations change the focus on sustainability. Kang et al., (2012) and Gössling et al., (2012) based their studies on the willingness of the consumers to pay them to green initiatives and the consumption of tourism resources, respectively, which laid early groundwork of environmental responsibility of hospitality. In a similar manner, Chan et al., (2014) & Chou, (2014) studied environmental beliefs and intentions of employees to engage in green practices and focused on human aspect of sustainability. New topics like green innovation and sustainability performance ( Asadi et al., 2020) and the increasing issue of overtourism ( Koens et al., 2018) indicate the growing complexity of the discipline. Moreover, De Grosbois, (2012) and Choi et al., (2015) helped to comprehend the idea of corporate social responsibility reporting and consumer decision-making in green hotels. All these works together show the theoretical richness and practical applicability of sustainability research in hospitality and tourism and they have great value as guides to researchers and industry practitioners.

**Table 4.** Most cited articles.

Authors	Article Title	Journal	TC
( Jiang & Wen, 2020)	Effects of COVID-19 on hotel marketing and management: a perspective article	International Journal of Contemporary Hospitality Management	495
( Kang et al., 2012)	Consumers’ willingness to pay for green initiatives of the hotel industry	International Journal of Hospitality Management	469
( Gössling et al., 2012)	Tourism and water use: Supply, demand, and security. An international review	Tourism Management	440
( Koens et al., 2018)	Is Overtourism Overused? Understanding the Impact of Tourism in a City Context	Sustainability	396
( Asadi et al., 2020)	Investigating influence of green innovation on sustainability performance: A case on Malaysian hotel industry	Journal of Cleaner Production	322
( Chou, 2014)	Hotels’ environmental policies and employee personal environmental beliefs: Interactions and outcomes	Tourism Management	318
( De Grosbois, 2012)	Corporate social responsibility reporting by the global hotel industry: Commitment, initiatives and performance	International Journal of Hospitality Management	314
( Choi et al., 2015)	Application of the extended VBN theory to understand consumers’ decisions about green hotels	International Journal of Hospitality Management	309
( Cater, 2006)	Playing with risk? participant perceptions of risk and management implications in adventure tourism	Tourism Management	271
( Chan et al., 2014)	What drives employees’ intentions to implement green practices in hotels? The role of knowledge, awareness, concern, and ecological behaviour	International Journal of Hospitality Management	271

TC = Total Citations

***4.2.5 Prolific authors***

At the top of the list of authors who have extensively written on the sustainability subject in the hotel and homestay industry is Han H., with 31 publications. Notably, Han H. also records the highest number of local citations (193), highlighting both the volume and impact of his contributions to the field. Hall C.M., a long-standing scholar in tourism sustainability, follows closely with 23 publications and 102 citations, reinforcing his influential role in shaping academic discourse as shown in Figure 5. Within the present pool of scholars, Lee S. has managed to produce 16–17 peer-reviewed articles and scored 116 citations, which means equally high scores in productivity and the potential audience of her scholarly work. A similar role is played by Elshaer I.A. (22 publications), Hassan T.H. (21), and Law R. (19), whose overall contribution has managed to expand the research in sustainable hospitality along several vectors of interest, as well as through closely related and thematically varied inquiries. Simultaneously, although they are not ranked in terms of publication volumes, when we consider the citation impact, Okumus F. (176 citations), Chan E.S.W. (166), and Gursoy D. (100) demonstrate a high citation impact, which means that the contribution of these scholars in their fields is significant and influential. The fact that these very prolific and highly referenced authors can exist at the same time signals growth in narrow specialisation and maturity of sustainability scholarship in hospitality and tourism ( Molina-Collado et al., 2022).

**Figure 5.**
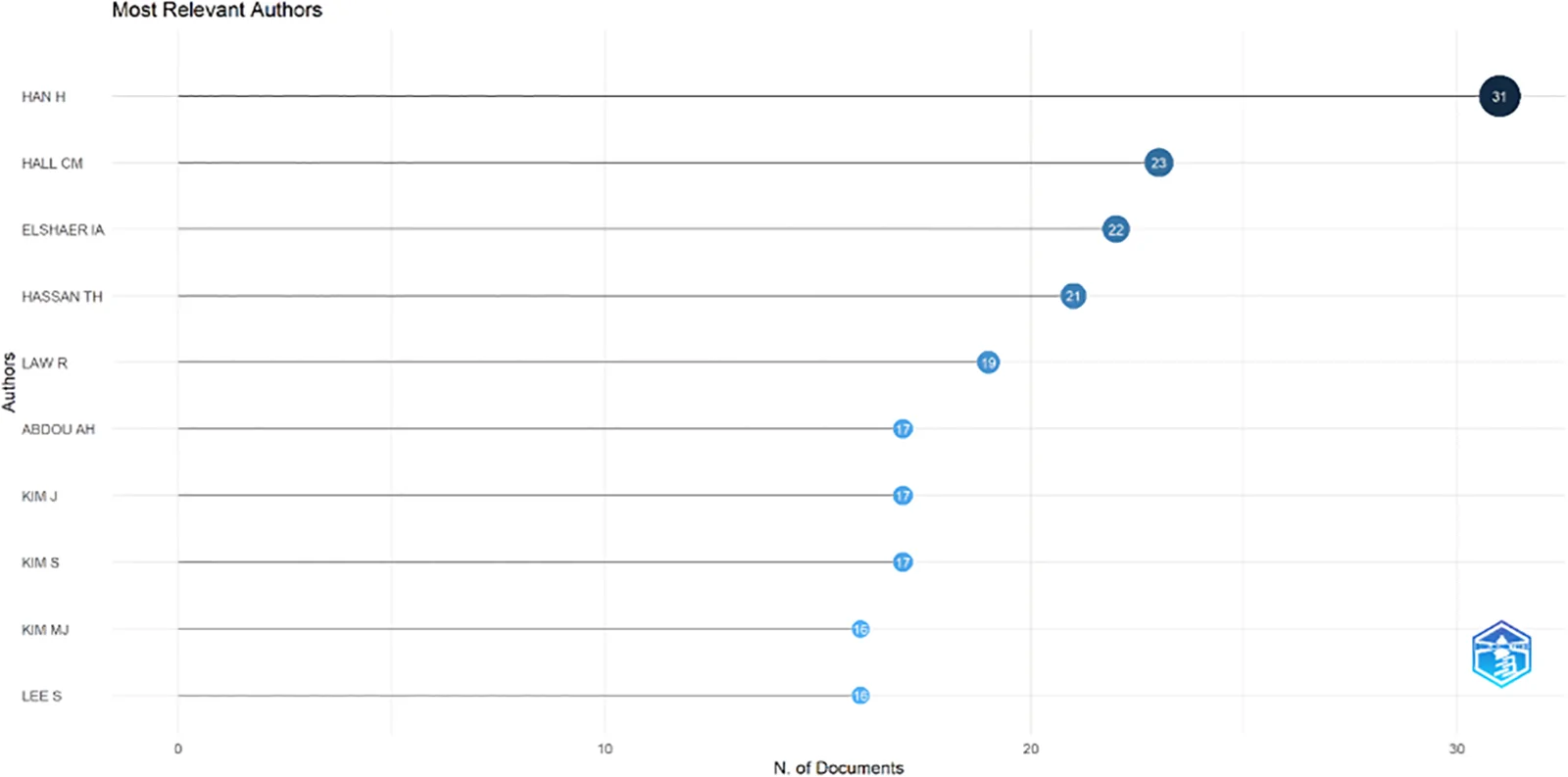
Prolific authors.

### 4.3. Conceptual and thematic structure

***4.3.1 The results of the co-occurrence analysis***

We analysed the most frequently used keywords in papers by researchers on the issue of sustainability in both hotels and homestays, aiming to gain a clear understanding of how they have approached the matter ( Aria & Cuccurullo, 2017; Donthu et al., 2021). A keyword co-occurrence map was generated using Biblioshiny, based on data extracted from the Web of Science Core Collection. In the visual output, the size of the circles represents the frequency of keyword occurrence, while the connecting lines indicate the strength of co-occurrence between terms.

The keywords that come out strongly in the centre include impact, satisfaction, tourism, management, and performance, and this reflects their relevance as meaningful anchors of sustainability studies in the hospitality industry as depicted in Figure 6. To illustrate the point, guest experience is at the heart of satisfaction because it is closely associated with quality, trust, and service. On the same note, management and tourism are inextricably linked to performance and behaviour, and researchers are interested in assessments of how sustainable practices influence the results of the organisation and the behaviour of the visitor. According to clustering, it is possible to see four large thematic groups:

**Figure 6.**
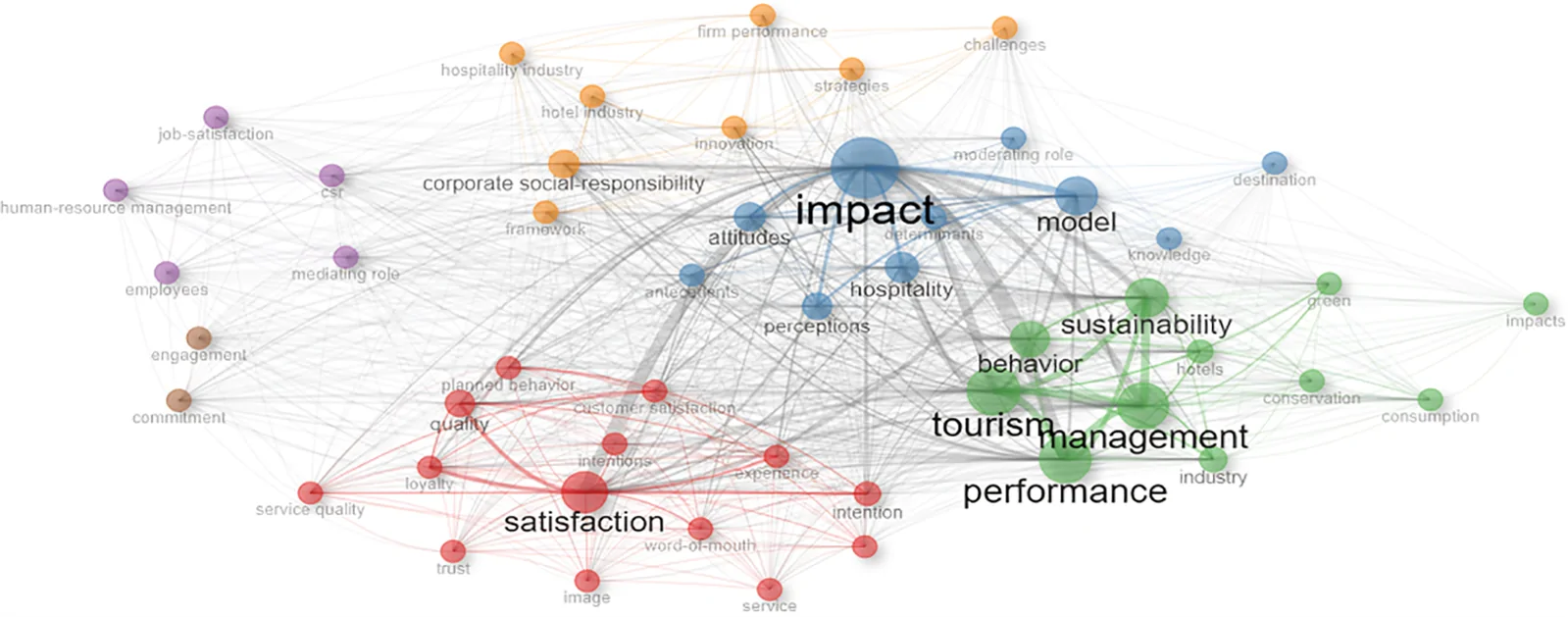
A network analysis of keyword co-occurrence.

**Cluster 1 (Red):** Service Quality and Customer Satisfaction. This cluster relates the quality, loyalty, trust, and service to satisfaction. It also brings out the importance of customer experience and service performance in developing sustainable hotel and homestay practices.

**Cluster 2 (Blue):** Impact and Perceptions. In this case, such keywords as impact, attitudes, perceptions, and hospitality intersect with each other with the mediating effect of perceptions and experiences in transforming sustainability outcomes.

**Cluster 3 (Green):** Tourism, Management, and Performance. This group of concepts connects tourism, management, sustainability, and performance by focusing on the operational and strategic aspects of sustainable hospitality practices.

**Cluster 4 (Orange/Purple):** CSR, Employees, and Innovation. That group, comprising corporate social responsibility, employee engagement, job satisfaction, and innovation, indicates the organisational issues and the importance of staff values in attaining sustainability.

On the whole, the keyword co-occurrence map reveals that even though sustainability research in hospitality is multi-dimensional, the main focus is still on guest satisfaction, impact assessment, and sustainable management, and other areas of emerging interest are CSR, employee engagement, and innovation.

For further clarity, Table 5 lists the top 10 most frequently used author keywords along with their total link strength, highlighting the predominant themes and conceptual linkages that shape the sustainability discourse within hotel and homestay research.

**Table 5.** Top 10 most frequent author keywords and their co-occurrence link strength in sustainability research on hotels and homestays.

Rank	Keyword	Occurrences	Total Link Strength
1	Sustainability	270	560
2	Tourism	129	313
3	Hospitality	105	280
4	Hotels	76	171
5	COVID-19	87	166
6	Sustainable Tourism	84	136
7	Hotel Industry	74	133
8	Corporate Social Responsibility	57	123
9	Hospitality Industry	51	108
10	Sustainable Development	51	91

***4.3.2 Thematic maps***

Figure 7 gives a thematic map that offers a detailed classification of the prevailing themes in the literature on sustainability in the hotel and homestay industry, which are combined in two dimensions: centrality (relevance) and density (development). The mapping provides a theoretical insight into current and upcoming directions in research contained in the field of sustainability discourse. The sustainability-behaviour-management-impact-satisfaction-model cluster is listed in the Basic Themes quadrant, which emphasises the support aspect of the discipline. The fact that it is the biggest among the four quads, as well as the fact that it is located in the centre of the quadrant, emphasises that the satisfaction of the guests, sustainability practices, and management impact are not merely crucial but also highly intertwined with the rest of the research clusters. It means that sustainability in hospitality is conceptualised more in terms of practical and result-focused. The attitudes-knowledge-personality cluster is placed in the Motor Themes quadrant, which refers to the fact that it is very central and dense.

**Figure 7.**
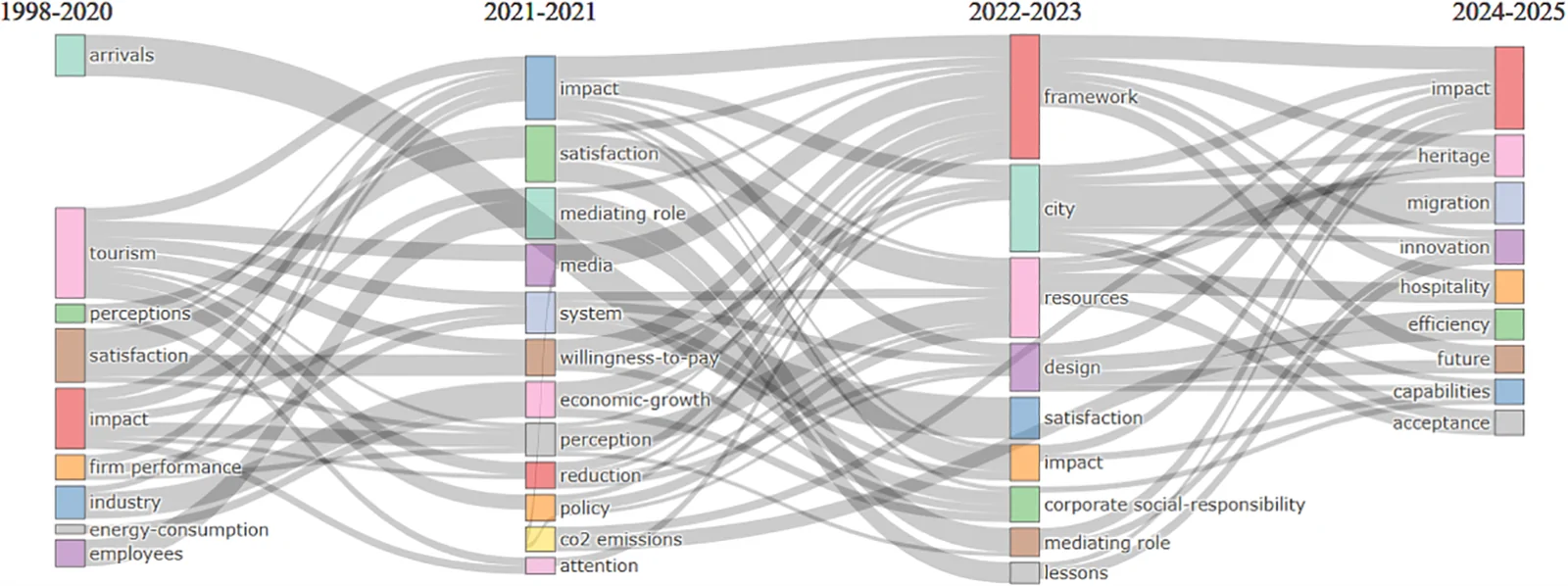
Thematic map in the context of sustainability research in homestays and hotels.

These themes are mature and powerful spheres of research, which emphasise the contribution of personal perceptions, behavioural patterns, and psychological factors to a sustainable hospitality practice. Conversely, the theme of energy consumption appears in the Emerging or Declining Themes quadrant with a fairly low centrality and density. This indicates that there is an existence of energy related researches, although they are insufficiently covered and are yet to be incorporated in mainstream discussions on sustainability. Nevertheless, there is great potential of this field in the future. Lastly, the Niche Themes quadrant has business models, hotel buildings, consumer acceptance and competitiveness. The fact that they are so dense and less central means that they are niche areas of study that are developed but slightly marginal to the larger sustainability literature. These themes are of academic rigour and niche development, especially in the treatment of innovativeness, operation strategy and consumer reaction towards sustainable hospitality. Altogether, the thematic map indicates that the literature prioritises the impact, the satisfaction, and the behavioural dimensions as the foundation of sustainability research, but, at the same time, points to the promising yet underutilised domains like energy consumption, governance, and business models that may determine the way research in hotels and homestays proceeds in the future.

***4.3.3 The results of thematic evolutionary analysis***

To investigate the advancement of thematic development in the sustainability research in hotel/homestay over the years, we explored the development of thematic change by visualising a Sankey diagram ( Aria & Cuccurullo, 2017), which is found in Figure 8. This image shows how the major research topics have changed and how they are interrelated with one another in four different time frames, including 1998–2020, 2021, 2022–2023, and 2024–2025. The themes are depicted with nodes, and the strength and continuity of such themes result in the thickness of the lines connecting the nodes. In the previous period (1998–2020), the basic terms or works touching on such aspects as tourism, satisfaction, impact, and perceptions prevailed. Moving to 2021, we see the emergence of more analytical and systemic-based themes like mediating role, economic growth, policy, and willingness-to-pay, meaning that sustainability has to study the drivers and recipients of sustainability. During the 2022–2023 phase, such themes as the so-called framework, resources, or corporate social responsibility and design were also on the rise, which indicates that an increased focus is placed on structured ways to employ sustainable practices. Lastly, in the latest segment (2024–2025), there has been an orientation to the newer terms, the heritages, migrations, innovation, and capabilities, which indicates a potential extension of the sphere of sustainability research to newer and more dynamic processes, such as cultural sustainability, human mobility, and efficient operations in the hospitality business. The prominent use of such themes as the impact and the satisfaction covering the same period also proves that these issues remain at the centre of the development of sustainable hospitality and homestay studies.

**Figure 8.**
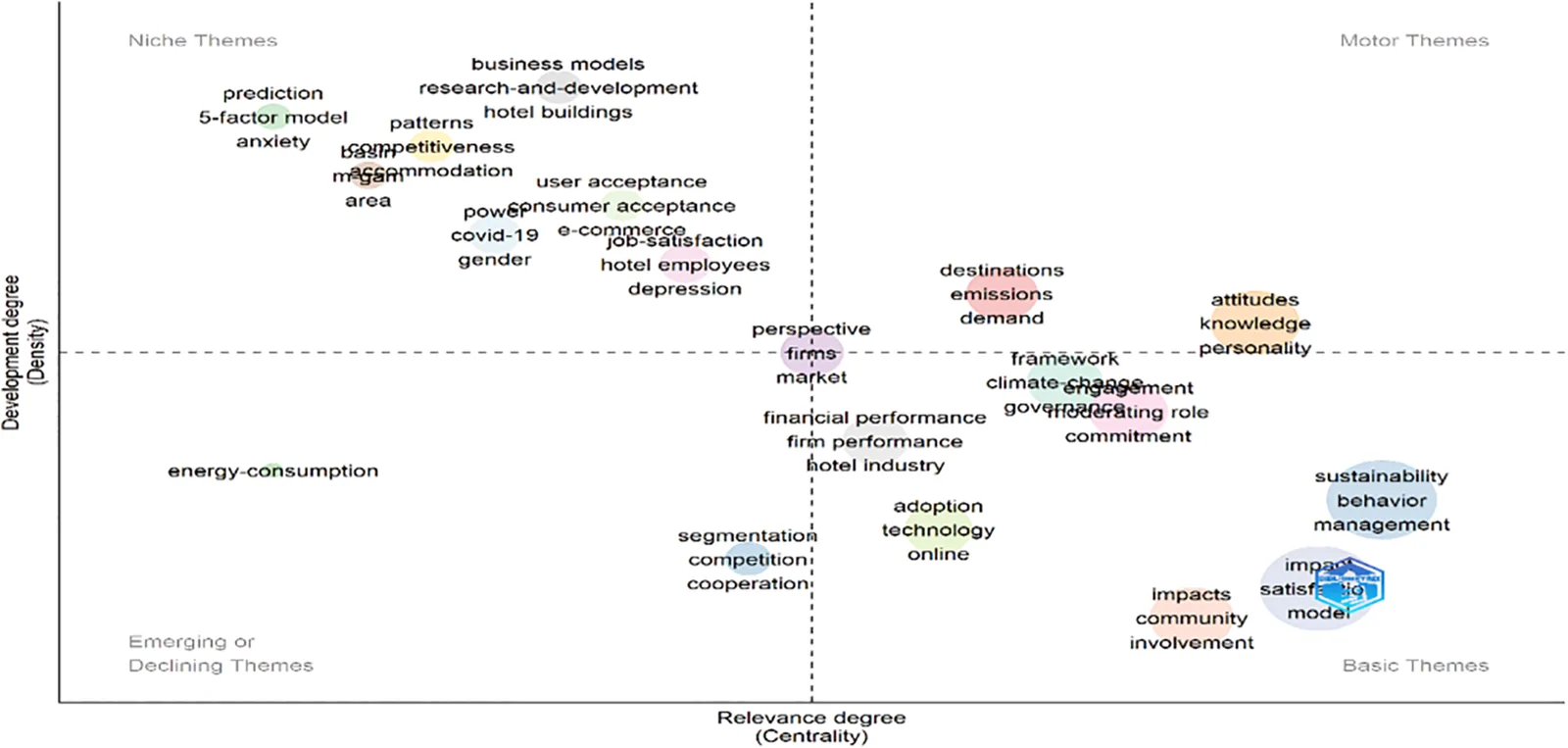
Thematic evolutionary analysis in the context of sustainability research in homestays and hotels.

### 4.4. Research gaps

The results of the thematic analysis ( Fig. 7) and of the thematic evolution mapping ( Fig. 8) further indicate a number of key gaps in the current state-of-the-art on sustainability in hotels and homestays. Although themes such as satisfaction of the guest, sustainable operations, and environmental responsibility are strongly represented, there are still several relevant ones that are inadequately explored or covered concerning theoretical integration and scholarly interest ( Horng et al., 2018; Janjua et al., 2021; Thakur et al., 2024). Thematic mapping revealed the presence of a number of low-density and low-centrality clusters (namely, those mapping to performance assessment, corporate social responsibility, and innovation) that apply to the periphery of the conceptual structure of the field. They are acknowledged yet barely intertwined with mainstream areas of focus in research, which implies that the themes have not attained a stage of conceptual and integration maturity in the mainstream discourse of sustainability in the hospitality sector ( Khatter, 2023; Molina-Collado et al., 2022). In addition, the thematic evolution analysis ( Fig. 8) shows the new, yet undeveloped areas like heritage, migration, and capabilities, which have only very lately started showing signs in academic discourse. Such themes demonstrate little consistency with previous periods of research and are not widely reflected in the present literature, which means that they could be examined in further detail ( Al-Hamad et al., 2024; De Bruyn et al., 2023; Geçikli et al., 2024). Besides those related to themes, we have gaps in contexts and methodology. The rural homestay, community-based strategies of sustainability, and technology-mediated sustainability plans are utilitarian fields that lack adequate research ( Krittayaruangroj et al., 2023). Furthermore, a methodological focus on quantitative designs has had minimal application of qualitative designs and mixed methods, designs that are vital in obtaining the views of the stakeholders, situational variability, and social dynamics ( Huang et al., 2025). These gaps need to be covered to further pursue a more interactive, diversified, and practically applicable research agenda in the field of sustainability in the hotel and homestay sectors.

### 4.6. Practical implications

The given research offers various implications to the stakeholders of the hospitality industry. To start with, the hotel and homestay administrators are in a position to optimise efficiency, cost reduction, and appeal to eco-conscious tourists by incorporating sustainable operations and environmental performance into their business strategies. The central clusters (guest satisfaction and employee engagement) indicate that the service providers must have a balanced approach between the ecological responsibility and the provision of quality services, and the well-being of the workforce. Hospitality businesses can become more competitive and, at the same time, address the expectations of the global standards of sustainability by implementing innovative solutions: energy-saving technologies, digital solutions to interact with guests, and environmentally friendly design. Second, the campaign of corporate responsibility can be extended to community development locally and to preservation of local cultures, so that sustainability extends beyond compliance to the creation of shared value. Policymakers will find the results valuable in the need to develop enabling policies and certification schemes that promote sustainable operations by both large hotels and small-scale homestays. Lastly, the thematic insights can be utilised by researchers and educators in training programs and curriculum development to equip upcoming professionals to handle the sustainability issues within the hospitality industry.

### 4.7. Theoretical implications

There are a few theoretical contributions made to the existing body of knowledge on sustainability in the hospitality industry in this study. To start with, the study has created a comprehensive framework linking service management, sustainability science and organisational studies through identifying six main clusters of research: guest satisfaction, sustainable operations, employee engagement, environmental performance, innovation and corporate responsibility. Such clusters emphasise the multidimensionality of sustainability and indicate that one theoretical framework alone cannot be used to understand its complexity. Second, the transformation of themes throughout the years reflects the theoretical change in the traditional models of service quality to more generalised models, which encompass the aspects of sustainability, corporate responsibility, and innovation. This shift is an indication of the increased relevance of interdisciplinary approaches, like institutional theory, resource-based view, and stakeholder theory, in explaining sustainability practices in hospitality. Third, the rise of more recent themes, such as the preservation of cultural heritage and technology-facilitated sustainability, is an indication of future potentials of theoretical development. Existing theories of sustainable hospitality may be extended with the help of adding the ideas of cultural studies, digital transformation, and behavioural science. On the whole, the results help to create a more developed theoretical framework that can be used by the researchers in future studies to observe sustainability in hotels and homestays in various cultural and geographic settings.

## 5. Conclusion and limitations

This paper gives a bibliometric analysis of the literature on sustainable practices in the hotel and homestay sector using 1,932 articles included in the Web of Science Core Collection. The analysis provides key thematic clusters, such as the areas of guest satisfaction, operational sustainability, and innovation, and also follows the development of the trends in research since 1998, using Bibliometrix and VOSviewer. Results show a growing academic interest since 2016 and demonstrate the progressive appearance of the topics of heritage, migration, collaboration of stakeholders, and technology-based sustainability practices. The knowledge helps to understand more clearly the intellectual framework and developmental path of the research in this field. The findings can guide the researchers, practitioners and policymakers to determine research streams that have a significant impact and those that have not been well studied in the hospitality industry.

The study has various limitations, regardless of its contributions. First, the sample was limited to publications in the English language, which were indexed in the Web of Science and may have omitted other databases like Scopus or non-English literature. Second, despite the fact that bibliometric tools, like VOSviewer and Biblioshiny, contribute to the transparency of the analysis, visual networks and thematic networks are still going to have their interpretation at the discretion of the researcher. Third, the research was based on a set keyword that included the terms related to sustainability, along with the terms hotel and homestay. This approach was able to guarantee the focus and consistency, but the relevant studies under other names (e.g., eco-lodges, guesthouses, community-based accommodation, or responsible tourism practices) could not have been identified. The research would be improved by using a wider or finer set of search terms in future studies to increase the coverage and inclusiveness. Lastly, the findings of this research can be expanded in case other databases are used, such as SCOPUS, Google Scholar and EBSCO.

### 5.1. Future directions

The thematic evolution analysis ( Fig. 8) could shed some valuable light on the future of the field of sustainability in hotels and homestays. Previous studies (1998–2020) mainly concentrated on the basic constructs, i.e. impact, satisfaction, and perceptions. Nonetheless, this has changed with recent developments (2021 and onwards) reflecting a turn toward broader and more interdisciplinary and context-specific themes ( Araña & León, 2021; De Bruyn et al., 2023; Shafiee et al., 2019). New emerging themes include heritage, migration, innovation and capabilities, which reflect a shift in the sustainability discourse. Despite having become visible, their depth and maturity are still insufficient, which implies that there is still a lot to discover in this field ( Richards, 2018). The future study can also investigate the role of sustainable practices in ensuring environmental and economic performance, as well as safeguarding local identity, architectural heritage, and intangible cultural resources ( Gomezelj, 2016; Richards, 2018). Research on culturally sensitive design and community-based tourism governance and host-guest relationships in the heritage setting may produce meaningful theoretical and practical implications ( Kontogeorgopoulos et al., 2015). The development of migration also underlines the rising importance of labour mobility, demographic changes, and host-guest relations in the context of hospitality sustainability ( Lugosi & Ndiuini, 2022). The causes of migration labour in sustainable hotel practices, potential effects of domestic migration on rural homestays, and social integration mechanisms within the local service ecosystem are some of the themes to be explored by future research ( Hack-Polay et al., 2022). Themes such as capability and innovativeness underline the strategic significance of organisational preparedness and technological change. Although it is gaining momentum, there is a lack of studies that deal with the application of smart resource management systems, energy-saving infrastructure, and digital guest engagement technologies ( Asadi et al., 2020; Pascual-Fernández et al., 2021). The future study may explore the mechanism of capacity building, the reasons behind the reluctance to adopt technologies, and the process of innovation diffusion among large-scale hotels and small homestayer businesses ( Gomezelj, 2016). Although rural homestays have the potential to promote inclusive and community-based sustainable development, they have not been theorised ( Kontogeorgopoulos et al., 2015; Ruhanen et al., 2015). There is a need to further seek more information on the stakeholder involvement, policy tools, infrastructure limitations, and the training mechanisms in geographically and economically marginalised areas. The current body of literature is largely quantitative in nature, methodologically. Mixed-method and qualitative research methods should be used in future studies to obtain the contextual and experiential aspects of integrating sustainability ( Pereira et al., 2021). The inclusion of the views of the hosts, guests, communities, and policymakers would give a more holistic view of the issues and solutions to sustainability. Altogether, the trend of the future scholarship ought to be in the direction of a more holistic, transdisciplinary, and context-sensitive perspective. The wider sustainability discussion, which incorporates the cultural, technological, social, and spatial aspects of sustainability in addition to the environmental and economic issues, will enhance theoretical elaboration and applicability in the research of hospitality. The major future research themes and corresponding research questions identified from this review are summarized in Table 6.

**Table 6.** Future research directions in sustainability research in hotels and homestays.

Proposed broader theme	Proposed research question
*Technology-enabled sustainability*	• How can smart technologies enhance resource efficiency in hotel and homestay operations?
	• What are the barriers to adopting digital tools for sustainability in small-scale accommodations?
	• How do digital guest engagement systems influence sustainable consumption behavior?
	• What role can automation and AI play in sustainable hospitality management?
	• How can mobile and IoT-based solutions improve real-time sustainability tracking in homestays?
*Cultural and heritage integration*	• How does the integration of cultural heritage in homestays contribute to sustainable development?
	• What are the challenges of preserving intangible cultural heritage in hospitality operations?
	• How can heritage-based homestays promote local identity while meeting sustainability goals?
	• In what ways do guests perceive and value cultural sustainability initiatives in homestays?
*Rural and community-based practices*	• What is the role of rural homestays in promoting equitable and community-led sustainable tourism?
	• How can local stakeholder engagement be strengthened in sustainability planning for rural accommodations?
	• What governance models support the scalability of sustainable homestay programs in rural settings?
	• How does infrastructure quality affect the sustainability performance of rural homestays?
*Stakeholder collaboration*	• What are the most effective stakeholder collaboration models in hotel sustainability strategies?
	• How do perceptions of sustainability differ among owners, staff, guests, and local communities in homestay contexts?
	• What incentives can drive cross-sector partnerships to promote sustainability in hospitality?
	• How does stakeholder communication influence the adoption of sustainable practices in hotels and homestays?
*Sustainability performance* *and innovation*	• How can innovation capabilities be measured and enhanced in sustainable hotel and homestay operations?
	• What frameworks exist for evaluating the sustainability performance of small hospitality businesses?
	• How do sustainability certifications impact operational behavior and guest perceptions?
	• What is the long-term impact of innovation-led sustainability programs in the accommodation sector?

## Data Availability

This work contains the following Underlying data Repository name: **Zenodo.** Title of project - Bibliometric Analysis of Sustainable Practices in Hotels and Homestays, https://doi.org/10.5281/zenodo.20374375 ( Saha & Singh, 2026). The project contains the following underlying data: Bibliometric Analysis of Sustainable Practices in Hotels and Homestays (Raw data). License: Data is available under Creative Commons Attribution 4.0 International. This work does not contain any Extended data.
