# Supplementary material for: Bibliometric Analysis of Sustainable Practices in Hotels and Homestays: Trends, Themes, and Future Research Directions
Source: F1000Res. 2026 Jul 6;15:1072. [Version 1] doi: 10.12688/f1000research.184973.1 (PMC13736066; doi:10.12688/f1000research.184973.1)
Supplement: Peer review history

# Reviewer report: Reviewer response for version 1

**DOI:** 10.5256/f1000research.204182.r534835

**Published:** 2026-11-04

**Authors:** Aruditya Jasrotia

**Affiliations:** 1 Amity University, Noida, Uttar Pradesh, India

The study addresses a timely and relevant topic by examining the evolution of sustainability research in hotels and homestays through a comprehensive bibliometric approach. The use of 1,932 publications from the Web of Science Core Collection, combined with Bibliometrix/Biblioshiny and VOSviewer, provides a strong foundation for identifying publication trends, influential contributions, thematic structures, and emerging research directions. The study's emphasis on rural homestays, corporate social responsibility, stakeholder collaboration, and technology-enabled sustainability offers valuable insights for researchers, practitioners, and policymakers. However, several aspects could be strengthened to enhance its academic rigour and practical contribution. The authors should provide a clearer justification for the database selection, search strategy, inclusion and exclusion criteria, and bibliometric indicators to ensure transparency and replicability. The distinction between hotels and homestays could also be explored more explicitly, particularly regarding their differing sustainability challenges and operational contexts. Furthermore, the identified research gaps would benefit from deeper critical discussion and more specific recommendations for future empirical and interdisciplinary research. Overall, this research makes a meaningful contribution to sustainable hospitality literature, and addressing these areas would further strengthen its clarity, methodological robustness, and scholarly impact.

Is the work clearly and accurately presented and does it cite the current literature?

Yes

If applicable, is the statistical analysis and its interpretation appropriate?

Yes

Are all the source data underlying the results available to ensure full reproducibility?

Partly

Is the study design appropriate and is the work technically sound?

Yes

Are the conclusions drawn adequately supported by the results?

Yes

Are sufficient details of methods and analysis provided to allow replication by others?

Yes

Reviewer Expertise:

Travel and Tourism Management

I confirm that I have read this submission and believe that I have an appropriate level of expertise to confirm that it is of an acceptable scientific standard.

# Reviewer report: Reviewer response for version 1

**DOI:** 10.5256/f1000research.204182.r534836

**Published:** 2026-11-04

**Authors:** Yashwant Singh Rawal

**Affiliations:** 1 Amity University Rajasthan, Jaipur, India

**Executive Summary**

 This manuscript presents a comprehensive bibliometric evaluation examining the intellectual, conceptual, and temporal evolution of sustainability research in hotels and homestays from 1998 to 2025. Utilizing 1,932 articles retrieved from the Web of Science Core Collection alongside Bibliometrix and VOSviewer tools, the authors map publication trajectories, influential authors, core journals, and thematic clusters. The study makes a notable contribution by differentiating broader hotel sustainability research from the underexplored niche of homestays, culminating in a forward-looking research agenda. The manuscript is well-structured and methodologically robust, though minor structural clarifications and methodological caveats will enhance its overall impact.

**1. Clarity, Accuracy, and Literature Review**

 The manuscript is clearly and logically structured, guiding the reader smoothly from the background rationale to data analysis and strategic implications. The terminology regarding bibliometric indicators (e.g., *h* -index, total link strength) is accurate, and the literature review properly situates the study within contemporary sustainability and hospitality discourse. However, the background section would benefit from a more explicit acknowledgment of potential database retrieval limitations regarding grey literature or alternative terminology (such as eco-lodges or guesthouses) earlier in the text rather than leaving it solely to the limitations section.

**2. Study Design and Technical Soundness**

 The research design is robust, employing established bibliometric protocols (SPAR-4-SLR principles and PRISMA guidelines) to ensure methodological transparency. The combination of Bibliometrix/Biblioshiny for conceptual mapping and VOSviewer for network visualization is standard, highly appropriate, and technically sound for large-scale bibliographic datasets.

**3. Methods and Replicability**

 The search strings, inclusion/exclusion criteria, and database filters (Web of Science Core Collection, English language restriction, article types) are transparently detailed, ensuring high replicability by future researchers. Furthermore, the underlying raw data has been made openly accessible via Zenodo, reinforcing the study's commitment to open science and reproducibility.

**4. Statistical Analysis (if applicable)**

 The study relies on bibliometric science mapping, citation metrics, and network analysis rather than traditional statistical hypothesis testing. This approach is standard and appropriate for science-mapping studies.

**5. Data Availability and Reproducibility**

 Complete transparency is maintained regarding data sources. The dataset of 1,932 records is fully archived and accessible on Zenodo under a Creative Commons Attribution 4.0 International license.

**6. Validity of Conclusions**

 The conclusions and future research directions directly stem from the bibliometric findings, thematic mapping quadrants, and Sankey diagram evolutionary analysis. The identified gaps—particularly concerning rural homestays, corporate social responsibility (CSR) integration, and stakeholder collaboration—are thoroughly supported by the quantitative evidence presented.

**Feedback & Recommendations**

**Major Revisions / Critical Points**

**1. Explicit Differentiation Between Hotels and Homestays in Discussion:** While the title and objectives frame the study around both *hotels* and *homestays*, the vast majority of the 1,932 indexed Web of Science papers heavily skew toward large-scale commercial hotels. The authors should expand the discussion to explicitly address how the bibliometric clusters reflect (or fail to reflect) unique homestay characteristics (e.g., family-run operations, community governance) versus corporate hotel chains.

**2. Methodological Scope Justification:** The search string deliberately combined general terms like "hotel" and "homestay" with sustainability keywords. Given that terms like "eco-lodges," "guesthouses," or "farmstays" are often utilized interchangeably in rural tourism literature, the authors should briefly address how omitting these synonyms might constrain the homestay-specific bibliometric retrieval.

**Minor Improvements / Clarity Edit**

**Formatting & Data Consistency:** Ensure uniform citation styles across all introductory paragraphs and verify that table callouts (e.g., Figure 1 through Figure 8, Table 1 through Table 6) align seamlessly with the narrative text.

**Abbreviation Expansion:** Define any specialized bibliometric terms (e.g., *h-index*, *SPAR-4-SLR*, *VOSviewer*) upon first mention to optimize readability for a broader hospitality management audience.

Is the work clearly and accurately presented and does it cite the current literature?

Yes

If applicable, is the statistical analysis and its interpretation appropriate?

Yes

Are all the source data underlying the results available to ensure full reproducibility?

Yes

Is the study design appropriate and is the work technically sound?

Yes

Are the conclusions drawn adequately supported by the results?

Yes

Are sufficient details of methods and analysis provided to allow replication by others?

Yes

Reviewer Expertise:

Sustanable Tourism, Tourist behavior, AI and Tourism, Tourism Marketing, Destination management

I confirm that I have read this submission and believe that I have an appropriate level of expertise to confirm that it is of an acceptable scientific standard.

# Reviewer report: Reviewer response for version 1

**DOI:** 10.5256/f1000research.204182.r534833

**Published:** 2026-11-02

**Authors:** Pravin Narayan Mahamuni

**Affiliations:** 1 Department of Business and Commerce, PSS Central Institute of Vocational Education NCERT, Bhopal, Madhya Pradesh, India

“The Bibliometric Analysis of Sustainable Practices in Hotels and Homestays: Trends, Themes, and Future Research Directions” manuscript reviews 1,932 articles from the Web of Science Core Collection spanning the period 1998–2025 about sustainable practices research in hotels and homestays. Authors used Bibliometrix/ Biblioshiny tool and VOSviewer for bibliometric network analysis and discussed several aspects related to publications, authors and journals influential in this area of research and productive institutions and countries along with collaborations patterns and thematic evolution regarding sustainability research conducted in hotels and homestays sector.

 The study addresses a relevant and evolving research area, considering the growing importance of environmental responsibility, sustainable business practices and community participation in hospitality management. The manuscript provides an overview of the development of scholarly interest in sustainability and identifies major research themes, including guest satisfaction, organizational performance, corporate social responsibility and innovation. It also highlights potential directions for future research, making the findings relevant to researchers and practitioners in hospitality and tourism. 

 Revisions may be required on the following:

1. Identify how your research will make a novel contribution to knowledge.
2. Increase Discussion Regarding Trends
3. Authors should identify how the results of their bibliometric analyses (i.e. keyword clusters, themes, emerging topics) led to the identification of these research opportunities.

The topic is interesting and useful. Sustainability studies involving hotels and homestays are important for the future of the planet. The methods and metrics used in the paper are appropriate. The data provided are sufficient to support the analysis and conclusions. Reading this paper provides the reader with a better understanding of the current literature including author output, highly cited publications, and topics of interest. Identifying topics for future research is beneficial to those researching within the hospitality/tourism industry. The authors provide the dataset used for their analysis which promotes transparency in research.

Is the work clearly and accurately presented and does it cite the current literature?

Yes

If applicable, is the statistical analysis and its interpretation appropriate?

Not applicable

Are all the source data underlying the results available to ensure full reproducibility?

Yes

Is the study design appropriate and is the work technically sound?

Yes

Are the conclusions drawn adequately supported by the results?

Yes

Are sufficient details of methods and analysis provided to allow replication by others?

Yes

Reviewer Expertise:

Business Management, Vocational /Skill Education, Online Learning/Education, Finance, MArketing

I confirm that I have read this submission and believe that I have an appropriate level of expertise to confirm that it is of an acceptable scientific standard.

# Reviewer report: Reviewer response for version 1

**DOI:** 10.5256/f1000research.204182.r503544

**Published:** 2026-07-28

**Authors:** Sharif Mohd.

**Affiliations:** 1 New Delhi Institute of Management, New Delhi, New Delhi, India

The manuscript, *"Bibliometric Analysis of Sustainable Practices in Hotels and Homestays: Trends, Themes, and Future Research Directions,"* addresses a timely and relevant topic within sustainable tourism and hospitality research. The bibliometric approach provides a useful overview of the evolution of research on sustainable practices in hotels and homestays and identifies emerging themes, influential authors, institutions, and collaboration networks. The study has the potential to serve as a valuable reference for researchers entering this field.

 The manuscript is generally well organised and easy to follow.

 However, several sections require further clarification and refinement before indexing. Strengthening the methodological description and providing greater transparency regarding the bibliometric procedures would significantly enhance the scientific contribution of the paper.

1. **Methodological Transparency: **The manuscript should provide a more detailed explanation of the bibliometric methodology. Please specify these details. They are essential for ensuring the reproducibility of the study.

- Date of data extraction.
- Inclusion and exclusion criteria.
- Document types considered.
- Language restrictions

2. **Database Justification**: If only one database (Web of Science) has been used, the authors should justify this choice. Including a discussion of database coverage would strengthen the methodological rigour.

**3. Interpretation Beyond Description**: While the bibliometric maps effectively describe publication trends and research clusters, the discussion remains largely descriptive. The manuscript would benefit from deeper interpretation of:

- Why certain themes have emerged.
- Factors driving publication growth.
- Practical implications for hospitality practitioners and policymakers.

**4. Future Research Directions**: The future research agenda is currently broad. Consider identifying more specific research opportunities, such as:

- AI-enabled sustainable hotel management.
- Carbon-neutral accommodation.
- ESG integration.
- Climate resilience.
- Sustainable digital tourism.
- Comparative studies between hotels and homestays.

Is the work clearly and accurately presented and does it cite the current literature?

Yes

If applicable, is the statistical analysis and its interpretation appropriate?

Not applicable

Are all the source data underlying the results available to ensure full reproducibility?

Partly

Is the study design appropriate and is the work technically sound?

Partly

Are the conclusions drawn adequately supported by the results?

Yes

Are sufficient details of methods and analysis provided to allow replication by others?

Yes

Reviewer Expertise:

Sustainable Finance, SDGs, Accounting and ESG

I confirm that I have read this submission and believe that I have an appropriate level of expertise to confirm that it is of an acceptable scientific standard, however I have significant reservations, as outlined above.
